# Monitoring Chronic Myeloid Leukemia: How Molecular Tools May Drive Therapeutic Approaches

**DOI:** 10.3389/fonc.2019.00833

**Published:** 2019-09-06

**Authors:** Barbara Izzo, Enrico Marco Gottardi, Santa Errichiello, Filomena Daraio, Claudia Baratè, Sara Galimberti

**Affiliations:** ^1^Department of Clinical Medicine and Surgery, Molecular Biology, University Federico II, Naples, Italy; ^2^Department of Clinical and Biological Sciences, University of Turin, Turin, Italy; ^3^Section of Hematology, Department of Clinical and Experimental Medicine, University of Pisa, Pisa, Italy

**Keywords:** CML, chronic myeloid leukemia, digital PCR, real-time PCR, NGS, mutations, ABL1, BCR-ABL1

## Abstract

More than 15 years ago, imatinib entered into the clinical practice as a “magic bullet”; from that point on, the prognosis of patients affected by chronic myeloid leukemia (CML) became comparable to that of aged-matched healthy subjects. The aims of treatment with tyrosine kinase inhibitors (TKIs) are for complete hematological response after 3 months of treatment, complete cytogenetic response after 6 months, and a reduction of the molecular disease of at least 3 logs after 12 months. Patients who do not reach their goal can switch to another TKI. Thus, the molecular monitoring of response is the main consideration of management of CML patients. Moreover, cases in deep and persistent molecular response can tempt the physician to interrupt treatment, and this “dream” is possible due to the quantitative PCR. After great international effort, today the BCR-ABL1 expression obtained in each laboratory is standardized and expressed as “international scale.” This aim has been reached after the establishment of the EUTOS program (in Europe) and the LabNet network (in Italy), the platforms where biologists meet clinicians. In the field of quantitative PCR, the digital PCR is now a new and promising, sensitive and accurate tool. Some authors reported that digital PCR is able to better classify patients in precise “molecular classes,” which could lead to a better identification of those cases that will benefit from the interruption of therapy. In addition, digital PCR can be used to identify a point mutation in the ABL1 domain, mutations that are often responsible for the TKI resistance. In the field of resistance, a prominent role is played by the NGS that enables identification of any mutation in ABL1 domain, even at sub-clonal levels. This manuscript reviews how the molecular tools can lead the management of CML patients, focusing on the more recent technical advances.

## Introduction

It is well-known that more than 95% of cases of chronic myeloid leukemia (CML) are characterized by the presence of the Philadelphia chromosome (Ph'), the deleted chromosome 22 produced by the reciprocal (but not fully balanced) translocation between the long arms of chromosome 9 and 22. During this event, two genes, Abelson 1 (*ABL1*), located on chromosome 9, and the Breakpoint Cluster Region (*BCR*), on chromosome 22, generate a fusion gene called *BCR-ABL1*, that, along with the Philadelphia chromosome, is the main diagnostic marker of CML (1).

Breakpoints within the *BCR* gene may be located in three different regions; in particular, the more common breakpoints are located downstream of exon 13 or exon 14 (e13, e14 named subtype b2 and b3), and more rarely (2–3%) on exons 6, 8, or 19 (2, 3).

**Figure 1:**
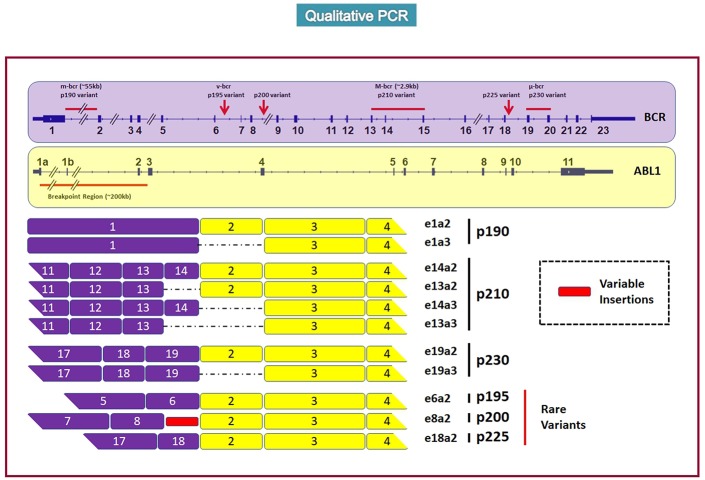
The figure represents the most frequent loci of rupture of the *BCR* and *ABL1* genes (up part) and the consequently origined proteins (P190, P210, P230, rare rearrangements) taken from Weerkamp et al. (7).

Breakpoints on the *ABL1* gene are located on exon 2, upstream (subtype a2) or downstream (subtype a3), with two different fusion constructs; both of these genes encode a protein of 210 kDa (p210). Alternatively, breakpoints might occur between exon 1 of *BCR* and exon 2 of *ABL1* (e1a2), thus encoding a protein of 190 kDa (p190), which is more frequently found in acute lymphoblastic leukemia (>75%), and more rarely in acute myeloid leukemia (2%) and in CML (<1%) (4, 5). Finally, the break downstream of exon 19 (e19-a2) can generate the “micro *BCR/ABL1*,” and its p230 protein, associated with a less aggressive “chronic neutrophilic leukemia” (6) (Figure 1).

BCR/ABL1 oncoproteins are constitutively active tyrosine kinases that promote the activation of different transduction pathway signals involved in cell growth and differentiation (*RAS, RAF, JUN, MYC, STAT, AKT*), able, therefore, to transform the hematopoietic stem cell in a neoplastic clone (8–11).

Many studies tried to describe the incidence of these different transcripts and to understand if proteins of different lengths might be responsible for different “phenotypes” of the disease. Recently, the prevalence of b2a2, b3a2 and the rare rearrangements have been assessed in more than 45,000 CML cases from 45 countries. b2a2 resulted to be the more frequent transcript (60%), followed by b3a2 (38%) and by “rare” rearrangements (occurring in only 2% of CML cases) (12).

The prognostic role of the different transcripts is still a matter of debate: in the scientific community, some authors support the hypothesis that b2a2 is associated with a lower rate of optimal responses (13–16), whereas other groups did not retrieve any prognostic differences (17). At the ASH meeting 2018, two different groups reported that the b3a2 form, compared to b2a2, seemed to be associated with a higher rate of deep molecular responses (DMR) (18) and of maintaining treatment-free-remission (TFR) (19).

**Figure 2:**
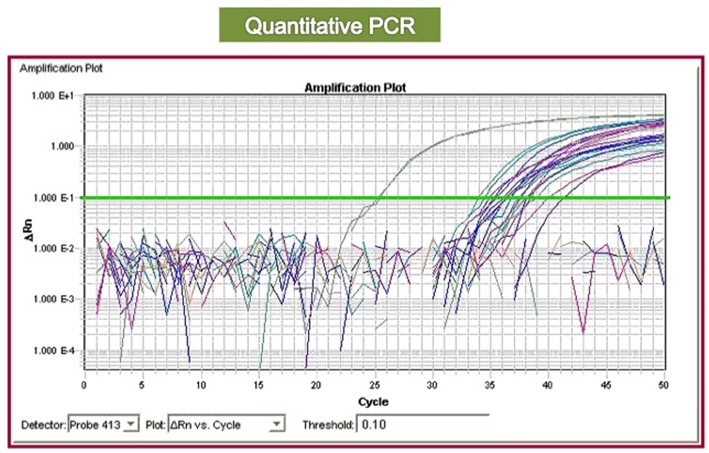
Quantitative PCR plot (RQ-PCR): the real-time amplification allows to measure the quantity of *BCR-ABL1* transcript by using a reference curve. By the measure of the threshold cycle (the cycle corresponding to the point where the amplification signal overcomes the background) is possible to calculate the concentration of each sample.

Qualitative PCR is useful for detecting *BCR-ABL1* and identifying the type of rearrangement (Figure 2); nevertheless, the type of encoded protein does not yet have a role in clinical practice, nor does it influence the choice of the first-line therapy. Conversely, the management of the CML patients is based on quantitative polymerase chain reaction (RQ-PCR), which allows us to stratify patients into “optimal responders” (who will continue the same treatment), “failed patients” (who need to immediately change therapy), and “warning cases” (who have to be closely followed to evaluate if and when to change the treatment), according to guidelines edited by the European Leukemia Network (ELN) (20, 21) or by the National Comprehensive Cancer Network NCCN (22).

## Real-Time Quantitative PCR: Its Fundamental Role in Management of CML Patients

Molecular monitoring plays an essential role in the clinical management of CML patients, leading the majority of clinical decisions.

After the introduction of the tyrosine kinase inhibitors (TKIs) in the treatment of CML, RQ-PCR became the gold standard to follow the disease burden reduction kinetics and to allow an accurate prognostic stratification.

In the IRIS trial (the study that allowed imatinib to enter into the clinical routine as a “magic bullet against cancer”), RQ-PCR showed that the reduction of the *BCR-ABL1/ABL1* ratio of at least 3 logarithms by 18 months represented an added value to the complete cytogenetic response (CCyR) in terms of long-term survival prediction (23). Interestingly, the “molecular load” significantly impacted on the 5-year event-free survival (EFS), which was 95% for cases in major molecular response (MMR = 3 logs of reduction) vs. 86% for cases with *BCR-ABL1/ABL1* ratio between 0.1 and 1%, 62% for patients with *BCR-ABL1/ABL1* ratio between 1% and 10%, and 58% for cases who still presented after 18 months a *BCR-ABL1/ABL1* ratio >10% (23, 24). Moreover, cases with a sub-optimal response at 18 months (no MMR) had a significantly higher risk of losing CCyR (24 vs. 0%) (25), thus highlighting the fundamental need of an accurate and reliable molecular monitoring.

The continuous therapeutic improvement of the last 10 years has led to increasingly ambitious treatment endpoints (now culminating in the possibility of achieving TFR), which, in turn, need more and more refined definitions of DMR levels, corresponding to a reduction of the *BCR-ABL1/ABL1* ratio of more than 4 logs (26, 27).

At an initial stage, the molecular quantitative approach was “coarse”: the 3 laboratories responsible for the molecular monitoring of patients enrolled in the IRIS trial, decided to mix 30 samples as “basal” (considered to carry 100% of the fusion gene); the reduction of the transcript was then measured from this value in logarithms (MR3 or MMR: reduction of *BCR-ABL1/ABL1* ratio of 3 logs = 0.1%; MR4: reduction of *BCR-ABL1/ABL1* ratio of 4 logs = 0.01%, MR4.5: reduction of *BCR-ABL1/ABL1* ratio of 4.5 logs = 0.0032%; MR5: reduction of *BCR-ABL1/ABL1* ratio of 5 logs = 0.001%) (28).

Moreover, until 2005 the quantification was still relative in the majority of labs: in fact, the transcript measured after 3 months of treatment was compared to the measurement initially obtained at diagnosis; the transcript was then measured after 6 months and compared to that of the third month and so on. It was a very difficult and time-consuming approach, especially for physicians. After that, the quantification became obsolete due to the introduction of a reference curve or of specific standards in the PCR reaction, which was definitely a success, but the standardization of the molecular tests was necessary to allow us to compare results deriving from different laboratories, thus introducing molecular response as the primary objective of the clinical trials.

In the scenario, in June 2007, collaboration between ELN and Novartis allowed the creation of the EUropean Treatment Outcome Study (EUTOS) consortium, with the purpose to create an international CML registry, standardize the molecular monitoring, design a path to cure, and become a network of excellence, able to optimize treatment of CML patients and to promote the cooperation among hematologists and scientists across all of Europe. Thirty-eight laboratories from 15 different countries began a process of methodological standardization of the *BCR-ABL1* measure and performed periodic quality controls (https://www.eutos.org/content/home/index_eng.html). Among these laboratories, 3 Italian centers were also present (Naples, Orbassano-Turin and Bologna), which started to spread the European project across Italy: in 2008, the Italian network, initially called “X-file,” and then “LabNet,” began its activity, with the aim of creating a network of molecular laboratories distributed throughout the national territory, which could manage the molecular response in CML, for the benefit of all Italian clinical centers. The project included a series of educational meetings, where the methodological aspects and the clinical significance of the evaluation of the residual disease were discussed and presented, with the purpose of rapidly expanding the importance of the molecular monitoring of CML in the TKIs era. We have to consider that at that time, although the prognostic role of the molecular response was evident, the technologies in use were still under discussion. The molecular investigations were carried out only in a few centers all over the world, and that monitoring was recommended to always be carried out by the same laboratory. In the same years, the methodological characteristics had been the subject of a “consensus conference” held in Washington in October 2005, where the technical characteristics were established for an optimal monitoring of the residual disease. A series of recommendations were discussed to harmonize the methodologies used in various laboratories by introducing a conversion factor (the International Scale, IS), which then became the standard to express results (29).

**Figure 3:**
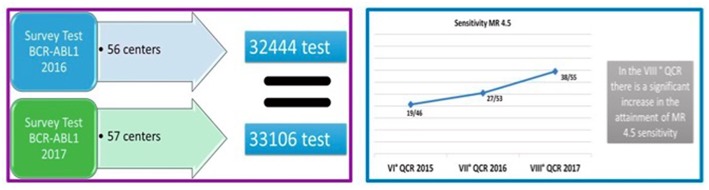
Increasing number of laboratories of the Italian LabNet network being able to attain a sensitivity level of MR 4.5 (*BCR-ABL1/ABL1* ratio = 0.0032%).

The Italian network initially started in 14 centers; at first it was decided to use a single methodology and a single technological platform in all laboratories, and the results clearly suggested that under controlled conditions the results were very reproducible, thus paving the way to the use of RQ-PCR in clinical routine. The network grew up with a dual purpose: to diffuse, in a progressively larger group of Italian laboratories, the new technologies, and to give diagnostic support in clinical studies designed by the Italian cooperative group “GIMEMA.” A dedicated software is used as a communication tool between clinical centers and laboratories, and a platform harvesting all regional databases on CML was created. Nowadays, LabNet consists of 55 laboratories that regularly participate in activities (such as training, methodological updating and quality controls) and constitute a valid and recognized diagnostic support for the whole country. Today, LabNet, thanks to constant development of its web platform, is the interface where clinicians and biologists collaborate in the management of the CML patients; moreover, LabNet allows clinicians to evaluate RQ-PCR as an accurate surrogate marker of response and is also a valid tool for assessing the patients' adherence to treatment. In 2017, as a result of the great effort of the Italian community, more than 33,000 RQ-PCR tests for *BCR-ABL1* have been performed, all laboratories were able to reach and maintain the MR4, and 69% of them attained the MR4.5, the backbone for a safe TFR (http://www.gimema.it/labnet-cml/) (Figure 3).

Currently, in the field of RQ-PCR, there remain some debated issues: (1) if and how strict the molecular monitoring should be to help physicians' decision in order to do the “early switch” (change of TKI in cases not reaching a *BCR-ABL1/ABL1* ratio ≤ 10% at 3 months); (2) whether or not the molecular techniques are really able to assess the stable DMR, a fundamental pre-requisite for TFR; 3) whether the definition of different “molecular classes” is reliable, which would allow physicians to offer TFR to the “optimal candidates” only, thus reducing the probability of TFR loss.

Concerning the role of the molecular monitoring by RQ-PCR and the “early switch,” the English group was the first one showing that *BCR-ABL1/ABL1* ratio at 3 months <9.84% was associated with a longer overall survival (OS) (8-year OS, 93% for cases with ratio <9.84 vs. 57% for those with a ratio >9.84%) (29). The German group found similar results and the CML IV study confirmed a significant difference in terms of 5-year OS between cases with *BCR-ABL1/ABL1* ratio lower or higher than 10% at 3 months (94 vs. 87%) (30). Interestingly, the predictive role of the Early Molecular Response (EMR), a reduction of at least 1 log in respect of diagnosis at 3 months of treatment, was effective not only for imatinib, but also when second-generation TKIs (dasatinib and nilotinib) were employed: in the ENESTnd trial, for example, where nilotinib was compared to imatinib as first-line treatment, EMR offered a clear advantage in terms of MR3 achievement and a lower probability of disease progression (31). The same results were obtained with dasatinib, both in terms of MR3 rate at 24 months (76% for cases with EMR vs. 16% for those with *BCR-ABL1/ABL1* ratio at 3 months >10%) and of 3-year OS (96 vs. 86%) (32).

Overall, the data reported above, even if retrospective, concur in sustaining the fundamental role of EMR; but what would happen to patients not in EMR if clinicians would wait for the molecular result at the 6 month instead of changing TKI just at the third month? The Canadian group measured the 3-year freedom from treatment failure rate for patients that at 3 months were not in EMR and who recovered or for patients who did not show the optimal response at the sixth month. The worst outcome was observed for cases with a transcript persistently >10% at the sixth month; nevertheless, no differences were observed between cases already “optimal” at 3 months and those who became “optimal” only after 6 months. This suggests that PCR values at 6 months might be more relevant than those from the third month, at least in the imatinib setting (33).

At the ASH meeting held in 2018, the matter of the “early switch” was discussed again: 108 patients in sub-optimal response or failing imatinib were early switched to nilotinib; the authors sustained that the early switch (change at the third month) offered a significant advantage in terms of MR3 at 2 years (78% of cases who switched to nilotinib after 3 months, 55% for those who switched between 3 and 12 months and 30% for patients who switched after 12 months or later) (34). At the same meeting, the results from the DASCERN trial were also presented, where patients not in EMR were randomized to switch to dasatinib at the third month or to continue imatinib; the MR3 rate at 12 months was significantly higher for cases who immediately switched to dasatinib (29 vs. 13%), even if the 3-year OS and progression-free survival (PFS) were the same in the two cohorts (35).

Some authors suggested as a possible solution to this debate the measure of the slope of the *BCR-ABL1* transcript reduction: indeed, in the group of cases with a *BCR-ABL1/ABL1* ratio >10% at 3 months, the outcome was better for those who showed a halving time <79 days, that could represent a favorable reduction kinetics that could prelude to the achievement of the optimal response at the second time-point (6 months) (36, 37). Similarly, another promising approach could be the measurement of *BCR-ABL1* transcript after only 4 weeks of treatment: in a series of 258 cases, the Receiver Operating Characteristic (ROC) curve showed that patients with a *BCR-ABL1/ABL1* ratio <41% after 1 month of therapy had higher probabilities of achieving the optimal response at 3 months, DMR (56 vs. 29%) and presented longer EFS (93 vs. 85%) (38).

Regarding the other two main issues—the ability of RQ-PCR to prove the stability of DMR, and the reliability of the correct definition of the “molecular classes,” we have to consider that, although RQ-PCR is always used to monitor the response to TKIs, it may not be the best approach when the issue is TFR followed by molecular relapse in half of patients who had profound and long-lasting molecular responses at the time of TKI discontinuation (39–41). In fact, to date, there is some evidence that we cannot accurately and reproducibly monitor those patients who are able to stay out of treatment indefinitely. This is probably due to several factors, such as the limit of detection of our molecular monitoring technology, or the inherent differences in the biology of leukemia (42), and/or immune response (43) in different patients, or a combination of several still undefined factors.

Indeed, among the methodological limitations of the RQ-PCR, there may be sampling errors, low sensitivity, or low/absent transcriptional levels of *BCR-ABL1* in the CML leukemic stem cell (LSC) that, when hidden in the hypoxic niche, does not synthetize the tyrosine kinase protein while it retains the fusion gene at DNA and RNA levels (44). For these reasons, researchers are always looking for increasingly sensitive techniques able to detect, with better accuracy and precision, the residual leukemic cells and to allow early identification of the patients who might be more likely to benefit from TFR.

The demonstration that the measure of the *BCR-ABL1* transcript is not sufficient to identify the perfect candidate for TFR treatment comes from a recent work that compared the number of LSCs measured in the peripheral blood by flow cytometry (as CD34+/CD38-/CD26+ population) with that measured by RQ-PCR. On a series of 400 patients, the absolute value of LSCs, either measured on peripheral blood or on bone marrow samples, did not correlate with the BCR-ABL1/ABL1 ratio measured by RQ-PCR in peripheral blood, with about 30% of patients with undetectable *BCR-ABL1* transcript levels still showing circulating CD26^+^ LSCs. Perhaps, molecular response could be considered as a picture of the transcriptionally active progenitor cells only (45).

Another “technical” hypothesis that could explain why half of patients rapidly lose TFR, is that there might be a mistake in categorizing patients; in other words, we would offer TFR to cases who are not in a real and persistent optimal response. This issue could perhaps be resolved by a new quantitative PCR, the digital PCR (dPCR).

## The Third Generation of Quantitative PCR: The Digital PCR

Several publications reported that dPCR is better at detecting MRD compared to RQ-PCR (46, 47), and that it provides an improved precision even at low *BCR-ABL1* transcript levels (46).

Today, dPCR represents one of the techniques that could be used for MRD monitoring, as it simplifies the standardization process and improves the sensitivity and precision of quantitative measurements. This method has already been employed in the hematological field by assessing mutations of *JAK2* (48), *B-RAF* (49), *DNMT3A* (50) or by quantitating some fusion genes, such as *PML-RAR*α (51) or *BCR-ABL1* (52) or by testing the rearrangement of the immunoglobulin heavy chain (*IGH*) (53).

In the classical RQ-PCR, an absolute standard curve with known amounts of *BCR-ABL1* is used to extrapolate the quantity of unknown samples by comparison between the amplification cycles of the standard references and of the samples (54). Nevertheless, the amplification may not always be perfectly efficient or variable, with, eventually, a lower sensitivity (55).

**Figure 4:**
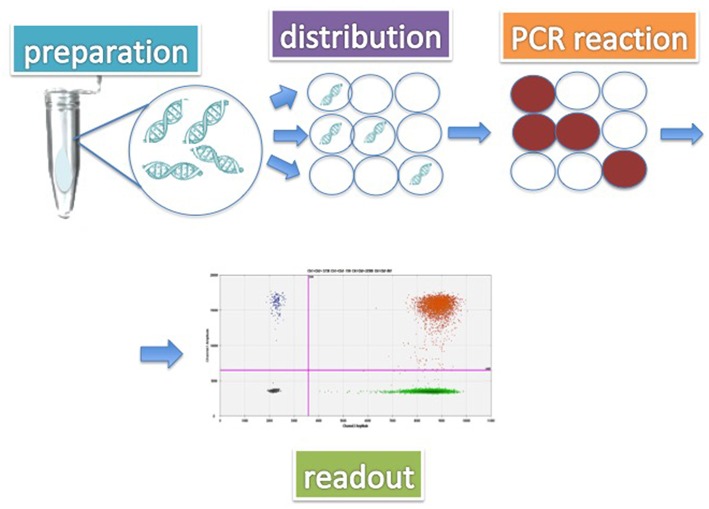
Digital PCR (dPCR) overview. The fundamental phases of the process are represented: (1) partitioning of the sample in thousand of drops; (2) amplification; (3) output and digital analysis.

dPCR offers an advantage over the conventional PCR, because samples are divided into thousands or millions of nanoliter reactions and amplification is performed in many spatially separated microscopic wells (the droplets), with higher efficiency (56) (Figure 4).

The purpose of dPCR is to have one template molecule in each partition; therefore, after amplification, each partition can be evaluated as “positive” or “negative” (digital reading). Consequently, the absolute quantity of molecules can be determined without the need for a standard curve. One of the main advantages of dPCR is the precise quantification of the nucleic acids, which facilitates the measurement of small percentage differences; overall, dPCR has the potential to detect the presence of the *BCR-ABL1* transcript with greater sensitivity and precision than RQ-PCR (57).

Several factors must be considered in the application of dPCR in CML: first, the “clinical” vs. the “analytical” sensitivity, which is expressed as the limit of detection (LoD) of an analyte, that indicates the lowest concentration detected with certainty at 95% of confidence interval (58). However, the “clinical” sensitivity of a test is defined as its ability to determine a log reduction of *BCR-ABL1*/*ABL1* ratio (or ratio between BCR-ABL1 and another reference gene) expressed internationally compared to the baseline. When “clinical” sensitivity is considered, three factors must be taken into account: (1) dPCR remains susceptible to pre-analytical errors upstream of the process, such as sampling, RNA extraction and cDNA synthesis, as already occurs for RQ-PCR; (2) the quantification of the reference gene is still necessary to evaluate the processing qualities of the sample and the pre-PCR phases; (3) the use of a conversion factor remains a fundamental requirement for the expression of results according to the international scale, which is, in turn, the basis for the classification of molecular responses.

As reported in other hematological malignancies, such as non-Hodgkin's lymphomas (59) and acute lymphoblastic leukemia (60), similarly in CML dPCR may be more sensitive than RQ-PCR, but it is always susceptible to the specific assay design, pre-PCR processing and molecular dropout (target not detected despite being present in the reaction). Moreover, positive and negative controls are needed to evaluate false negative and false positive samples and to define the quantification thresholds.

**Figure 5:**
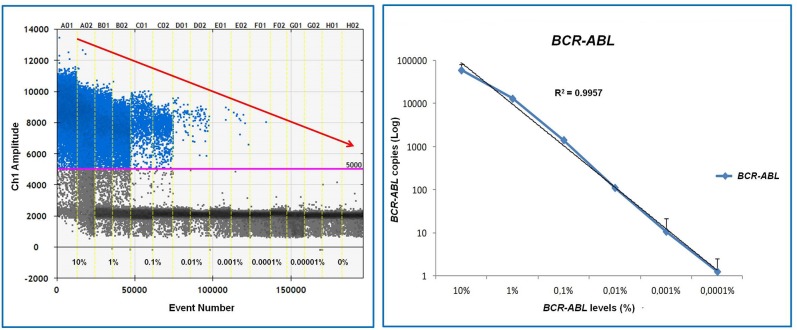
Assessment of detection performance of dPCR system in RNA samples obtained from serial dilutions of Ph'+ cells in Ph'-cells. As shown, dPCR is able to detect *BCR-ABL1* mRNA up to 0.001% (MR5).

The application of dPCR for the molecular monitoring in CML is a growing area of research: initial studies using both nanofluidic approach and dPCR showed that dPCR can measure transcript below 0.01% (46, 47), but not without some false negative results. An interesting study reported a robust quantification of *BCR-ABL1* levels from 0.0032% (MR4.5) to 10% across three different dPCR platforms: Bio-Rad QX200, RainDance RainDrop System, and Applied Biosystems QuantStudio3D. This study successfully detected *BCR-ABL1* down to MR5, thus demonstrating that dPCR approach was able to increase sensitivity detection levels (61) (Figure 5).

However, another study reported that *BCR-ABL1* values between MR3 and MR5 generated by the Bio-Rad QX200 system were consistently higher than values generated by conventional RQ-PCR (62). Nevertheless, the concordance between dPCR and RQ-PCR was good, with values between 89 and 97%, according to the employed platform (61). Other authors confirmed the high sensitivity of dPCR: in the MR4.5 class, 76% of cases resulting negative by RQ-PCR were still positive by dPCR when the cut off for positivity was set at 3 droplets (63, 64). Moreover, at the European Hematology Association (EHA) meeting held in 2018, it has been reported that 16% of the cases enrolled in the EURO SKI trial, who did not show any *BCR-ABL1* transcript when QR-PCR was performed on cDNA, resulted *BCR-ABL1*-positive when dPCR was performed on genomic DNA. Therefore, leaving the question unanswered as to what would be the best informative compartment for MRD assessment in CML (65).

Similarly, in another series of 230 patients enrolled in the ENEST first trial, 10% of cases defined as in MR4 by RQ-PCR resulted in MR3 by dPCR (62); this induces to hypothesize that at least one quarter of CML cases who lost TFR were effectively cases that had not been adequately scored by RQ-PCR. Different groups tried to identify a specific *BCR-ABL1* value that would be significantly associated to the TFR loss; Nicolini et al. showed that the loss of TFR was conditioned by the duration of treatment with Imatinib (longer than 75 months was favorable) and by a cut off of 0.023% (measured by dPCR) (66). Another group reported that the probability of losing TFR was 53% in the cohort of cases, showing *BCR-ABL1/ABL1* ratio >0.468 vs. 17% in the subgroup where the transcript values were <0.468 (67). Finally, age higher than 45 years and positive d-PCR at the moment of imatinib discontinuation were the two factors able to condition the loss of TFR in the ISAV study (68).

In conclusion, d-PCR seems to be a good, novel, sensitive and accurate tool for the molecular monitoring in CML; nevertheless, an international standardization is necessary before this technique enters routine molecular methods.

## Next Generation Sequencing (NGS) and its Use in CML

The scenario of the new molecular technologies employed in CML has been recently enriched by the Next Generation Sequencing (NGS); this method is now frequently used for detecting *ABL1* kinase domain (KD) mutations, that are one relevant mechanism of CML resistance to TKIs and that account for ~50% of acquired resistance in failing CML cases (69). Indeed, many different single KD mutations have been associated with resistance to imatinib, but also to nilotinib (Y253H, E255K, E255V, F359V, and F359C), dasatinib (V299L, T315A, F317L, F317I, F317V, and F317C) or bosutinib (Y253H, V299L, F317V) (70–77).

Even if these TKI-specific mutations are present in <10% of cases who do not reach the optimal response, it is relevant to identify and characterize them, in order to evaluate what kind of TKI has to be employed at the moment of the switch for resistance.

**Figure 6:**
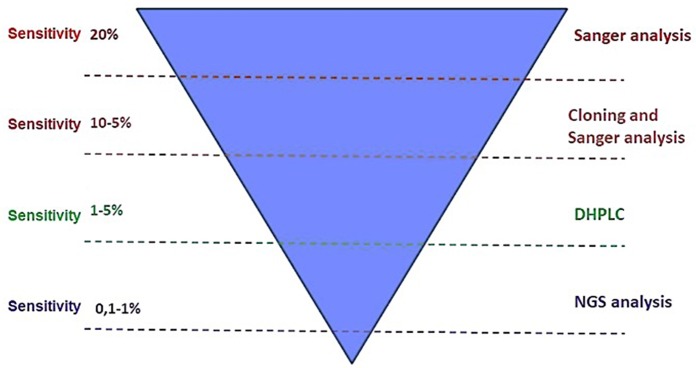
Sensitivity of different molecular biology methods for *ABL1* kinase domain mutations detection: the deepest sensitivity is reached by Next Generation Sequencing (NGS) tools.

*ABL1* KD mutations are commonly detected by sequencing *BCR-ABL1* cDNA according to the Sanger's method. This technique, however, can only detect mutations present in a percentage higher than 10–20% (78) (Figure 6). Other techniques with greater sensitivity, such as High-Performance Liquid Chromatography (HPLC) (78) and mass-spectrometry (79) can identify mutations with a minimal mutation load <1% (Figure 4). In 2004, Soverini et al. (78) developed a new HPLC-based assay that they compared to the conventional Sanger. Both D-HPLC and Sanger detected *ABL1* mutations in 12/27 patients; nevertheless, 2 cases, that have been scored as wild-type by Sanger, resulted mutated by HPLC. In 2010, Placzek et al. (79) set a novel biosensor in a cellular model for measuring *ABL1* kinase activity; this sensitive method represented a new tool for mutational screening. Indeed, it was able to identify mutations by measuring *ABL1* kinase activity that resulted reduced in mutated vs. the wild-type samples.

The “NGS” definition collectively refers to some high-throughput methods that can sequence large numbers of RNA or DNA molecules and can be digitally tabulated. These platforms result in very high numbers of sequencing reads and allow for discovery of sequences occurring at lower frequencies, even when observed in a heterogeneous background. An important feature of this technology is the massive quantity of information generated, and consequently, sophisticated bioinformatics and computational methods are required for analysis. Compared to the Sanger sequencing, which is characterized by a limit of sensitivity of approximately 10–20%, NGS reaches sensitivities of 1–10% and may reveal a complex clonal architecture consisting of a dynamic mix of polyclonal and compound mutations (80–82).

NGS includes two different strategies: (1) the “target capture” via probe hybridization or ligation, and (2) the “amplicon based enrichment” (ultra-deep sequencing [UDS] or the “amplicon” deep sequencing [ADS]). Hybridization-based NGS uses synthetic oligonucleotides specifically designed to target *BCR* and *ABL1*, followed by sequencing. Fusion junctions are predicted using bio-informatic packages designed to identify structural variants, including chromosome translocations, amplifications, inversions and deletions. These packages usually produce one or both types of reads: (1) split reads, that are single reads composed of material from two non-contiguous genomic regions directly mapping a fusion junction to a base pair resolution; and (2) discordant pairs of reads, in which there are individual reads in a pair map to different chromosome locations, indicating the presence of a structural rearrangement within the insert between them. When a sample has an expression level of *BCR-ABL1* >10%, a mean read-depth of 50x is sufficient to map the fusion junction. The mapped genomic fusion junctions of each patient are unique and can be used as patient–specific markers for MRD monitoring if DNA or RNA at diagnosis are available. Amplicon deep-sequencing method utilizes a highly multiplexed amplicon generation strategy where multiple regions of interest are amplified and then sequenced at a depth of 100–10,000-fold greater than that offered by Sanger sequencing. Dedicated softwares assemble, align and map the sequenced reads to the reference sequence and perform variant detection. The quantitative feature is gained by sequencing targeted regions at depths of hundreds or thousands of reads, allowing the sensitive detection and quantification of rare events. ADS has been used for sensitive quantification of *ABL1* mutations (83–86); overlapping primers designed to cover the KD within the fusion *BCR-ABL1* transcript were used to amplify the domain by a nested PCR approach to enrich it for the fusion transcript. Amplicons from multiple samples are barcoded and clonally amplified for sequencing. Sequencing the amplicons on a high throughput platform allows sufficient depth of coverage (≥2,000 reads) per base to identify mutations at very high sensitivity (<1%).The application of amplicon deep sequencing for monitoring the genomic fusion junctions as a molecular marker is also a plausible quantification approach that is usually used to detect and track clonally-expanded lymphocytic populations in acute lymphoblastic leukemia (87, 88) and other malignancies (89–93). Whether amplicon deep-sequencing can compete with the sensitivity achieved by quantitative methods, such as RQ-PCR or d-PCR, is still unknown.

From the clinical point of view, it seems that *ABL1* mutations could also be responsible for resistance when present at a low level, and that detection of multiple KD mutations is associated with an inferior response rate compared to patients with none or with single mutations (94).

Compound mutations (more than one mutation in a single molecule) have also emerged as a potential driver of acquired resistance to first- and second-generation TKIs, and show different resistance profiles compared with individual mutations, some of them also being resistant to ponatinib, the third-generation TKI able to overcome the negative prognostic impact of the majority of the *ABL1* mutations, including the T315I (95). Compound mutations being resistant to ponatinib is still matter of debate: indeed, some authors sustain that compound mutations are always resistant (96, 97), whereas, others have demonstrated that only few combinations are really resistant to ponatinib. In particular, at the last ASH meeting, Soverini et al. (98) showed that compound mutations characterize only 3.5% of the chronic phases, 32% of the accelerate/blastic cases and 37% of cases progressed into Ph'-positive acute lymphoblastic leukemia. The most accurate and sensitive molecular method for detecting these compound mutations seems to be NGS; however, some studies conveyed that sequencing artifacts and PCR recombination can also lead to some, even if rare, false-positive compound mutations, with a possible over-estimation (99).

On the other hand, novel advances and refinements of the NGS-based sequencing approaches can be interesting: for example, the incorporation of unique molecular identifier tags into the NGS protocol demonstrated that sequencing errors can be identified and removed from analysis (94), resulting in the ability to distinguish compound mutations from PCR-artifacts or polyclonal mutations (different mutations on different CML cells). Thus, Deininger et al. (83) devised an algorithm to predict false-positive compound-mutation: this algorithm was based on the recombination rate, which was determined by measuring the frequency at which false-compound mutations were generated when RNA samples from two patients, each with already known distinct single *ABL1* mutations, were mixed. Only compound mutations with an observed frequency greater than the false-compound mutation rate were considered “true” compound mutations.

Notwithstanding NGS is not yet fully standardized, as previously occurred for the RQ-PCR, an international attempt of harmonization has been performed in the last few years: the IRON II study was conducted by a consortium of 10 laboratories from 8 countries engaged in the standardization and validation of a common protocol for *ABL1* mutation screening: a concordance between Sanger and NGS was found in 394/398 of the tested samples (100). As for the LabNet, this international project was exported to Italy, where a network of 4 laboratories (Bologna, Catania, Orbassano, Napoli) sharing a common NGS protocol and an optimized pipeline of data analysis was created and shared. With collaboration of 39 Italian clinical centers, 211 “failing” patients were assessed: NGS was able to detect a mutation in 45% of them, in comparison with 20% who resulted mutated by Sanger, and 36% of cases showed “low-burden” mutations. The most relevant finding was that 24% of patients already scored as “wild-type” by Sanger, resulted mutated by NGS. Furthermore, NGS found that mutations showed a clinically relevant profile for the choice of TKI, thus supporting a relevant clinical role for NGS in CML (101).

## Conclusions

For many years, the assessment of MRD has been fundamental for better managing of treatment of CML patients with TKIs, and this process has been done in an easier way than in other hematological malignancies because the fundamental pathogenetic mechanism of this disease has been well clarified in the last few years, making the *BCR-ABL1* transcript the principal target of all MRD tests (102).

The technique that is commonly adopted for measuring the *BCR-ABL1* transcript is the quantitative PCR, which could be performed either by real-time or dPCR; the new published data have shown that dPCR could be more sensitive and accurate than RQ-PCR and, in future, we'll see if dPCR will replace RQ-PCR in the CML context. Moreover, the best target nucleic acid (mRNA or DNA) is still matter of debate, because it has been demonstrated that LSCs also persist in patients achieving DMR and during TFR. The availability of new molecular methods will probably provide the answer to this question.

Another hot field in the CML scenario is represented by the adoption of the NGS tools for evaluating the mutations of the *ABL1* kinase domain, these being the mutations responsible for resistance to TKIs either in the chronic or in more advanced disease phases. Until now, the Sanger sequencing has been the more commonly adopted method, but NGS, with its higher sensitivity, is able to detect mutations at a sub-clonal level as well as compound mutations that are responsible for the resistance to ponatinib.

The use of NGS will likely be more frequently adopted for patients who fail the first line of therapy and for those who are in suboptimal response during further treatment lines. In the allogeneic transplantation setting, data produced with NGS are not yet available and Sanger is still the recommended technique.

Overall, in this review we have demonstrated the potential to manage many and new promising molecular tools that can help physicians to design, *ab initio*, a successful and patient-tailored treatment. Obviously, further harmonization of these molecular techniques by international projects is still necessary to speak a universal language.

It is clear that, the basis of successful management of CML patients will always be the strict collaboration between biologists, technicians, and physicians. The existing and future networks will help all of us to reach this goal.

## Author Contributions

BI, EG, and SG wrote the manuscript. SE and FD revised the part of methodologies. CB revised the clinical parts.

### Conflict of Interest Statement

The authors declare that the research was conducted in the absence of any commercial or financial relationships that could be construed as a potential conflict of interest.
